# Anticancer and Multidrug Resistance-Reversal Effects of Solanidine Analogs Synthetized from Pregnadienolone Acetate

**DOI:** 10.3390/molecules19022061

**Published:** 2014-02-17

**Authors:** István Zupkó, Judit Molnár, Borbála Réthy, Renáta Minorics, Éva Frank, János Wölfling, Joseph Molnár, Imre Ocsovszki, Zeki Topcu, Tamás Bitó, László G. Puskás

**Affiliations:** 1 Department of Pharmacodynamics and Biopharmacy, University of Szeged, Eötvös u. 6, Szeged 6720, Hungary; E-Mails: molnarjudit85@freemail.hu (J.M.); rethyborbala@pharm.u-szeged.hu (B.R.); minorics@pharm.u-szeged.hu (R.M.); 2 Department of Organic Chemistry, University of Szeged, Dóm tér 8, Szeged 6720, Hungary; E-Mails: frank@chem.u-szeged.hu (É.F.); wolfling@chem.u-szeged.hu (J.W.); 3 Institute of Microbiology and Immunology, University of Szeged, Dóm tér 10, Szeged 6720, Hungary; E-Mail: molnar.jozsef@med.u-szeged.hu; 4 Department of Biochemistry, University of Szeged, Dóm tér 9, Szeged 6720, Hungary; E-Mail: imre@bioch.szote.u-szeged.hu; 5 Department of Pharmaceutical Biotechnology, Faculty of Pharmacy, Ege University, Bornova, Izmir 35100, Turkey; E-Mail: zeki.topcu@ege.edu.tr; 6 Department of Obstetrics and Gynaecology, University of Szeged, Semmelweis u. 1, Szeged 6725, Hungary; E-Mail: bito.tamas@med.u-szeged.hu; 7 Laboratory of Functional Genomics, Institute of Genetics, Biological Research Center, Hungarian Academy of Sciences, Temesvári krt. 62, Szeged 6726, Hungary; E-Mail: puskas.szbk@gmail.com; 8 Avidin Ltd., Alsókikötő sor 11, Szeged 6726, Hungary

**Keywords:** solanidine analog, anticancer action, efflux pump

## Abstract

A set of solanidine analogs with antiproliferative properties were recently synthetized from pregnadienolone acetate, which occurs in Nature. The aim of the present study was an *in vitro* characterization of their antiproliferative action and an investigation of their multidrug resistance-reversal activity on cancer cells. Six of the compounds elicited the accumulation of a hypodiploid population of HeLa cells, indicating their apoptosis-inducing character, and another one caused cell cycle arrest at the G2/M phase. The most effective agents inhibited the activity of topoisomerase I, as evidenced by plasmid supercoil relaxation assays. One of the most potent analogs down-regulated the expression of cell-cycle related genes at the mRNA level, including tumor necrosis factor alpha and S-phase kinase-associated protein 2, and induced growth arrest and DNA damage protein 45 alpha. Some of the investigated compounds inhibited the ABCB1 transporter and caused rhodamine-123 accumulation in murine lymphoma cells transfected by human MDR1 gene, expressing the efflux pump (L5178). One of the most active agents in this aspect potentiated the antiproliferative action of doxorubicin without substantial intrinsic cytostatic capacity. The current results indicate that the modified solanidine skeleton is a suitable substrate for the rational design and synthesis of further innovative drug candidates with anticancer activities.

## 1. Introduction

Molecules with a steroidal skeleton exert an exceptionally wide range of pharmacological activities, despite their highly conserved chemical structures. Besides the well-characterized hormonal actions (e.g., sexual steroids, gluco- and mineralocorticoids), many steroids isolated from plants exert a broad variety of pharmacological actions, including antioxidant, neuroprotective, antihypercholesterolemic and ionotropic properties [1,2,3,4,5]. Antiproliferative activities are among the most intensively investigated properties of natural products and their synthetic analogs; the importance of such studies may be illustrated by the fact that nearly 80% of all the recently approved anticancer agents are either natural products or their synthetic analogs [6]. Cardiotonic steroids (e.g., digoxin and digitoxin) are traditionally utilized in cardiovascular medicine and their pronounced antiproliferative activities were recently recognized in retrospective epidemiological analyses. During the last decade, intensive research has paved the way for their further development and their application for cancer prevention or treatment [7]. A continuously increasing number of chemically diverse natural steroidal products with antiproliferative activities have been isolated, but most do not have substantial effects on the classical hormonal receptors [8]. The most promising of these natural compounds have been used for the design and synthesis of a wide variety of novel analogs with a view to understanding the structure–activity relationships in order to optimize the structure. Steroidal alkaloids are characteristic secondary metabolites commonly found in species of the Solanaceae, Apocynaceae, Liliaceae and Dioscorea families. The antiproliferative capacities of the most abundant molecules, including diosgenin, solasodine and tomatidine, against human cancer lines are well characterized [9]. Locally applied solasodine glycosides have been investigated in a double-blind, randomized and placebo-controlled clinical study on patients with basal cell carcinoma. The cream was found to be effective in the histologically confirmed clearance of the cancer cells, and 78% of the patients had had no recurrence at the 1-year follow-up [10]. In a separate subset of innovative steroid-based anticancer agents, the steroid backbone has been utilized as a carrier and linked to a cytotoxic pharmacophore (e.g., a nitrogen mustard moiety). This type of drug candidate is typically designed for targeting of estrogen- and androgen-dependent cancers [11]. 

In a project designed to find novel lead-candidates, androstane-based analogs were designed and their antiproliferative properties were screened [3,12,13,14,15]. A set of solanidine analogs were synthetized from pregnadienolone acetate (which occurs in Nature) and subjected to a preliminary study of their cytostatic activities against human adherent cancer cell lines [15,16]. The starting material of the synthesis was recently produced from an extract of the fermentation broth of an endophytic fungus, *Sphaceloma* sp. LN-15, isolated from the leaves of *Melia azedarach* L. and cultivated in pure culture [17]. The aim of the present study was the characterization of the antiproliferative action of these novel solanidine analogs. Their proposed mechanisms of action were approximated by means of a set of *in vitro* experiments including flow-cytometric cell cycle analyses, plasmid supercoil relaxation assays and RNA profiling with nanocapillary, quantitative real-time PCR (QRT-PCR). 

Multidrug resistance (MDR), which is usually manifested as a consequence of the overexpression of ATP-binding cassette (ABC) efflux transporters, is one of the greatest challenges in the development of anticancer drugs. Inhibition of such efflux pumps may be regarded as an approach to reverse MDR and therefore restore the efficacy of the utilized anticancer drugs [18]. ABCB1 (also known as P-glycoprotein or P-gp), is frequently involved in MDR is the most intensively studied transporter of this class. Since chemically related steroidal compounds have been reported to inhibit the ABCB1 efflux pump, the rhodamine-123 exclusion assay was used in order to extend our investigations to the MDR-reversal properties of the current set of solanidine analogs [19]. 

## 2. Results and Discussion

### 2.1. Cell Cycle Analysis

**Figure 1:**
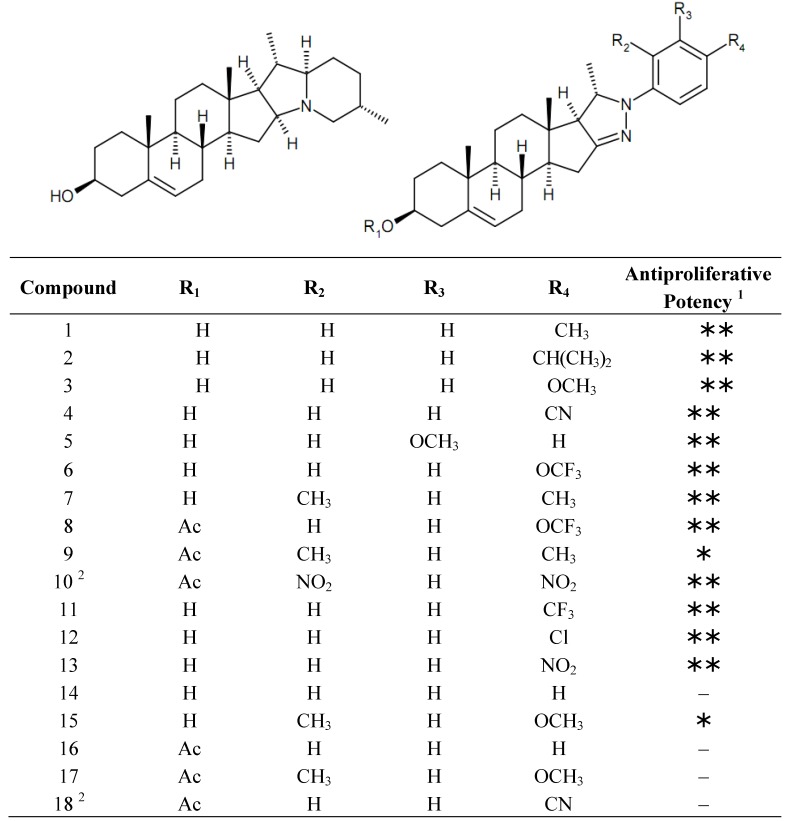
Chemical structures of solanidine (**left**) and the tested analogs (**right**).

^1^ Calculated IC_50_ values against HeLa cells are below 10 µM (∗∗), between 10 and 30 µM (∗) or above 30 µM (–). For exact values see [16]; ^2^ Containing a pyrazolidine ring instead of a 2-pyrazoline.

The chemical structures of the currently investigated set of solanidine analogs are presented in Figure 1. 

**Figure 2:**
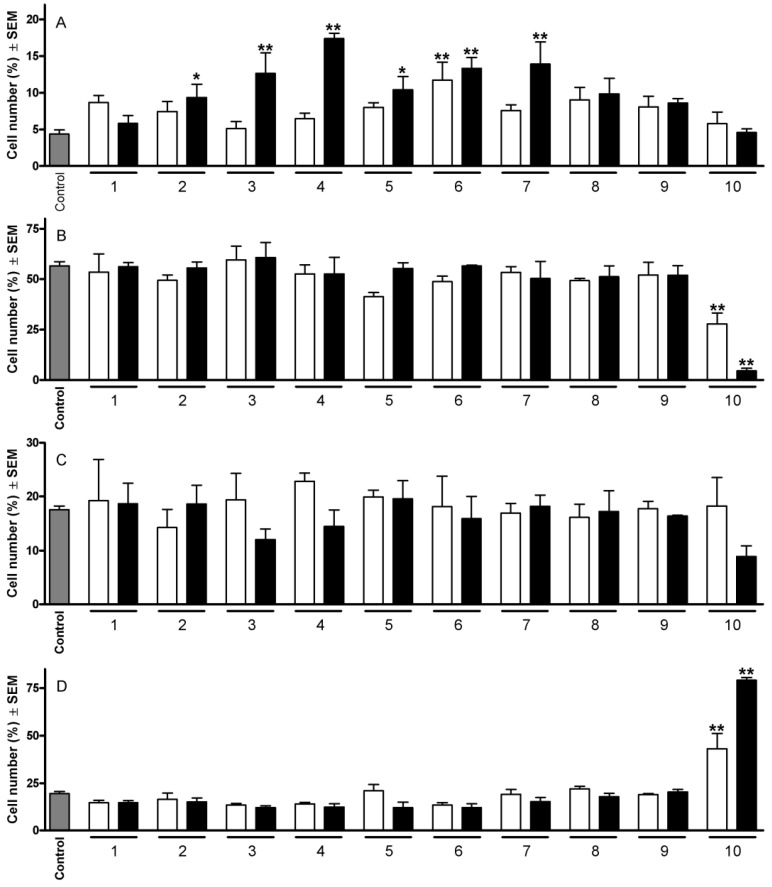
Effects of **1**–**10** on the HeLa cell cycle distribution after 24 h at 10 (open columns) and 30 µM (filled columns). Panels A, B, C and D indicate cells in subG1, G1, S and G2/M phase, respectively. * and ** indicate *p* < 0.05 and *p* < 0.001, respectively, as compared with the control cells.

On the basis of previous evaluations of their antiproliferative potencies, 10 of them were selected for flow-cytometric cell cycle analyses at two concentrations (10 and 30 µM). Treatment of HeLa cells with compounds that had previously exhibited IC_50_ values < 10 µM generally elicited a profound change in the cell cycle distribution (Figure 2). The most outstanding action detected was the substantial increase in the hypodiploid (subG1) population. In this respect, compound **6** was the most potent one, causing a significant elevation of the hypodiploid population even at the lower concentration. Comparison of the results obtained with pairs of molecules differing only in the substituents at position 3 (*i.e.*, pairs **6** and **8**; **7** and **9**) revealed that a hydroxy group is more favorable than an acetoxy function. The other cell cycle phases were not substantially disturbed by the treatment with the tested compounds, with the exception of **10**, which elicited a cell cycle arrest as indicated by a concentration-dependent decrease in the G1 population and an increase in the G2/M cells.

### 2.2. Plasmid Supercoil Relaxation Assay

**Figure 3:**
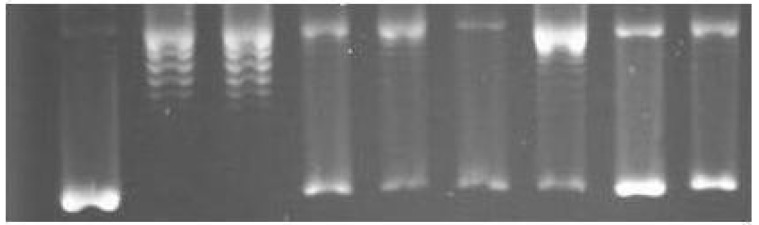
The effects of the solanidine analogs on mammalian DNA topoisomerase I activity. A representative agarose gel photograph of supercoil relaxation in the presence of 0.1 mM concentrations of the test substances. Lane 1, pBR322; lane 2, pBR322 with 1 u of DNA topoisomerase I; lane 3, same as lane 2 with DMSO, lanes 4 to 9, pBR322 with 1 u DNA topoisomerase I in the presence of 0.1 mM of **14**, **12**, **3**, **1**, **4** and **2**, respectively.

Supercoil relaxation assay screening of some of the selected test compounds involved monitoring as discrete bands for supercoiled (sc) and relaxed (rlx) DNA separated in gel electrophoresis. Inhibition of the relaxation activity was detected in the form of sc DNA, migrating faster than rlx DNA (Form I). A representative gel analysis of the supercoil relaxation activity of DNA topoisomerase I in the presence of 0.1 mM of the compounds is given in Figure 3. The relaxation of the sc plasmid substrate, pBR322, by topoisomerase I (Figure 3, lane 2) was not influenced by the presence of DMSO (Figure 3, lane 3), which was used for the dissolution of the test compounds. However, relaxation of sc pBR322, was profoundly affected upon incubation with **3**, **4** and **14**, as evidenced by the average 65%, 61% and 56% of sc pBR322 remaining, respectively, in the form of Form I DNA, obtained from the relative band intensities of Form I and Form II DNA (Figure 3). Among the other test compounds, **2** and **12** exerted intermediate interference, while **1** had only a negligible effect on the relaxing ability of the enzyme (Figure 3). Residual Form II DNA, seen in the sc pBR322 preparation, was taken into consideration by subtracting from the band intensity corresponding to the relaxation of the enzyme for each test compound. The lower concentrations of the test compounds gave rise to a decreased band intensity of Form I DNA, with an accumulation as slower-migrating Form II DNA, demonstrating the concentration-dependent nature of the effects (data not shown).

### 2.3. RNA Profiling with High-Throughput Nanocapillary QRT-PCR

To study the effects of the different steroid compounds, it seemed appropriate to focus on a subset of genes that may serve screening purposes for specific cell cycle markers. The aim of the present study was to analyze the effects of different steroids at different concentrations and time points with multiple repetitions, and we therefore applied a customized nanocapillary QRT-PCR platform to profile cell cycle- and apoptosis-related genes in response to drug intervention. Accordingly, from the DNA-microarray and literature data we selected 56 genes that cover different pathways which undergo changes during the cell cycle and which have roles in apoptosis; cyclin-dependent protein kinases (CDC2, CDK2, CCNH, CCND1, cyclin E2, CKS1B and CKS2), DNA and chromatin binders (MCM3,5, PCNA, BRCA1 and BRCA2), DNA repair (HUS1, GADD45a and RAD17), TNF and death receptors (TNFRSF1B, TNFRSF4, TNF, TNFRSF10A and TNFRSF10B), transcription factors (EGR1, TP73, FOXOA3, CDKN2A, CDKN2B, CCNT1 and CCNT2), kinases (EGFR, ABL1, DAPK2 and DAPK3) and ubiquitin-dependent proteins (UBE1, APC2, APC4 and SKP2). The list of the selected genes, together with their functional categories and primer sequences, can be seen in Supplementary Table 1.

Two analogs (**10** and **16**, which are one of the most effective and ineffective, respectively) were applied at three different concentrations (10 μM, 20 μM and 30 μM) and their effects on the cell cycle-related gene expression were determined at three time points (6 h, 12 h and 24 h following treatment). Only eight of the selected 56 genes were observed to exhibit changes in expression (Table 1). At 6 h, only one gene displayed a changed expression: cyclin-dependent kinase inhibitor 2B (CDKN2B) was induced 2.27-fold in the case of **16** at the highest dose, but this change proved to be only just at the significance level. At 12 h, only two genes exhibited differential expression, with compound **10** down-regulating the expression of tumor necrosis factor alpha (TNFα) in a concentration-dependent manner, which suggested not only cytostatic, but also anti-inflammatory action, while both **10** and **16** slightly induced the expression of S-phase kinase-associated protein 2 (SKP2) at 30 μM. Interestingly, at 24 h the expression of the same gene was dramatically decreased in response to treatment with **10**. One of the key players regulating cell cycle progression is the F-box protein SKP2. Its protein level is regulated during the cell cycle. Moreover, the overexpression of SKP2 is associated with a variety of human cancers, indicating that SKP2 may contribute to their development [20,21]. The down-regulation of SKP2 has been correlated with the induction of apoptosis in lung-cancer cells [22].

**Table 1:** QRT-PCR analysis of cell cycle related genes in response to drug treatment.

		**10**								
		**6-h incubation**			**12-h incubation**			**24-h incubation**		
**GeneName**	**RefSeqID**	**10 M**	**20 M**	**30** **M**	**10 M**	**20 M**	**30 M**	**10 M**	**20 M**	**30 M**
TNFα; tumor necrosis factor alpha	NM_000594	−1.02	−1.08	−1.23	1.51	−3.90	−8.41	1.72	−1.87	−4.00
GADD45alpha	NM_001924	1.59	1.04	1.24	1.16	1.28	1.72	1.14	5.19	3.65
TXNRD1; thioredoxin reductase 1	NM_003330	1.24	−1.73	−1.11	−1.40	−1.39	−1.42	1.78	1.90	1.28
SKP2; S-phase kinase-assoc. prot. 2	NM_005983	1.87	−1.55	−1.43	−1.06	−1.53	2.11	−4.58	−7.60	−8.04
APC4; anaphase-promoting complex 4	NM_013367	1.31	1.25	1.22	1.27	1.45	1.03	−2.45	−2.16	−2.23
CCND1; cyclin D1	NM_053056	−1.42	1.05	−1.04	1.25	1.57	−1.30	−1.43	−2.14	−4.36
CCNE2; cyclin E2	NM_057749	1.04	−1.12	1.09	1.24	1.23	−1.08	1.23	1.13	−1.23
CDKN2B; cyclin-dependent kinase inhib. 2B	NM_078487	−1.22	−1.26	1.27	1.05	−1.50	1.02	−2.74	−7.11	−9.36
		**16**								
		**6-h incubation**			**12-h incubation**			**24-h incubation**		
**GeneName**	**RefSeqID**	**10 M**	**20 M**	**30 M**	**10 M**	**20 M**	**30 M**	**10 M**	**20 M**	**30 M**
TNFα; tumor necrosis factor alpha	NM_000594	1.56	-1.76	−1.69	−1.13	−1.72	−1.75	−2.23	1.16	1.45
GADD45alpha	NM_001924	−1.02	1.12	1.27	1.67	1.38	−1.07	1.78	1.86	2.63
TXNRD1; thioredoxin reductase 1	NM_003330	1.01	1.14	1.01	1.68	−1.18	1.37	2.42	2.01	2.60
SKP2; S-phase kinase-assoc. prot. 2	NM_005983	1.03	−1.08	1.67	1.74	1.34	2.10	−1.40	−1.87	−1.30
APC4; anaphase-promoting complex 4	NM_013367	−1.10	1.26	1.43	1.75	1.08	1.11	−1.28	−1.90	−1.43
CCND1; cyclin D1	NM_053056	1.15	1.11	1.63	1.07	1.22	1.09	−1.60	−1.01	−1.19
CCNE2; cyclin E2	NM_057749	1.09	1.30	1.48	1.94	1.44	1.83	−1.33	−2.62	−2.33
CDKN2B; cyclin-dependent kinase inhib. 2B	NM_078487	1.69	1.18	2.27	-1.61	1.96	1.88	−6.22	−7.86	−14.83

After a 24-h incubation, the up-regulation of the expression of thioredoxin reductase 1 (TXNRD1) by **16** indicated the induction of oxidative stress and the related defense mechanism by the cell [23]. Both of the steroids were observed to induce growth arrest and DNA damage protein 45 alpha (GADD45 alpha). The GADD45 family of proteins, which includes α-, β-, and γ-isoforms, has recently been shown to play roles in apoptosis, the DNA damage response and cell cycle arrest. Moreover, the expression of either GADD45alpha or GADD45gamma activated the P38 and JNK kinase pathways and induced G2/M arrest [24].

### 2.4. Rhodamine-123 Accumulation Assay

The effects of the solanidine analogs on the intracellular accumulation of rhodamine-123 were investigated at two concentrations (40 and 400 μM) on a mouse lymphoma cell line (L5178) expressing the functional ABCB1 transporter (Table 2). From the perspective of the transporter-inhibiting property, the functionality at position 3 appears crucial, as reflected by the lower activities of the acetylated analogs (compounds **8**, **9**, **16**, **17**) relative to those of the hydroxy congeners (compounds **6**, **7**, **14**, **15**). Of these analogs, **4**, **5**, **13** and **15** proved to have the most efficient MDR-reversal effects, resulting in a more than 100-fold increase in rhodamine-123 accumulation with at least one of the applied concentrations. On the other hand, compounds **2**, **8** and **17** exerted no effect on the pump function. Some of the compounds (e.g., **14** and **15**) influenced the accumulation of the marker with an apparently inversely proportional concentration–effect relationship. The exact mechanism of this behavior is beyond the scope of the present study, but toxicity at the higher concentration seems a plausible explanation. 

**Table 2:** Effects of solanidine analogs on the rhodamine-123 accumulation assay in L5178 MDR mouse lymphoma cells. Each datum is the mean ± SEM of the results of three experiments.

Compound	Concentration (µM)	Fluorescence activity ratio		Compound	Concentration (µM)	Fluorescence activity ratio	
1	40	3.71 ± 0.96		11	40	4.72 ± 0.53	
	400	50.15 ± 7.62			400	84.03 ± 14.22	
2	40	1.38 ± 0.11		12	40	28.74 ± 8.31	
	400	6.86 ± 0.96			400	49.07 ± 19.09	
3	40	40.68 ± 13.82		13	40	2.52 ± 0.06	
					400	187.6 ± 51.67	
	400	2.27 ± 0.37					
4	40	169.6 ± 43.77		14	40	36.31 ± 9.56	
	400	106.1 ± 17.11			400	14.33 ± 1.20	
5	40	51.60 ± 12.57		15	40	135.1 ± 48.35	
	400	114.4 ± 26.51			400	4.58 ± 0.58	
6	40	2.91 ± 1.76		16	40	12.25 ± 3.66	
	400	10.16 ± 2.77			400	50.24 ± 3.75	
7	40	30.59 ± 10.05		17	40	1.72 ± 0.46	
	400	44.90 ± 16.08			400	7.27 ± 2.30	
8	40	1.62 ± 0.15		18	40	6.74 ± 1.70	
	400	2.82 ± 0.94			400	21.98 ± 4.33	
9	40	3.16 ± 1.34		Verapamil	40.6	5.93 ± 1.92	
	400	17.15 ± 3.67					

### 2.5. Combination Experiments

**Figure 4:**
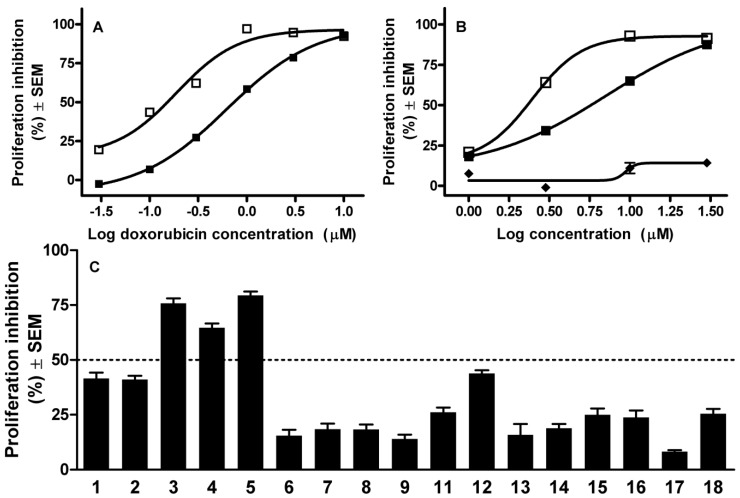
(**A**) Antiproliferative action of doxorubicin against parental (□) and MDR (■) mouse lymphoma cells; (**B**) Antiproliferative actions of **3** (□), **4** (■) and **5** (♦) against MDR mouse lymphoma cells; and (**C**) Antiproliferative effects of the solanidine analogs (**1**–**9** and **11**–**18**, 5 µM) combined with 0.1 µM doxorubicin.

In order to obtain further information concerning the potential importance of the ABCB1-inhibiting capacity of the compounds, combination experiments were performed with doxorubicin, a substrate of the pump. The antiproliferative potency of doxorubicin against both transfected and parental mouse lymphoma cells was determined by means of the MTT assay, which indicated IC_50_ values of 0.75 and 0.15 μM, respectively, revealing that the ABCB1 transporter confers resistance against the substrate (Figure 4, panel A). A minimally effective concentration of doxorubicin was selected for combination with the currently tested solanidine analogs in a 72-h MTT assay. The overall action of 0.1 μM doxorubicin (which alone elicits ~7% inhibition of the proliferation of transfected cells) in combination with 5 μM of the analogs is presented in Figure 4, panel C. Three of the tested steroids that most efficiently inhibited the ABCB1 pump substantially increased the antiproliferative action of doxorubicin. In theory, this potentiating action may be attributed to the MDR-reversal properties of the steroids or their direct cytostatic action. To answer this, direct antiproliferative actions of these three substances were evaluated under the same conditions (Figure 4, panel B). Two of the selected compounds, **3** and **4**, proved to exert intrinsic antiproliferative activities with calculated IC_50_ values of 1.71 and 5.73 µM, respectively, while **5** did not exhibit any substantial action on the growth of the treated cells.

### 2.6. Discussion

There is increasing evidence of the anticancer potential of both natural and synthetic steroidal molecules. We have presented here *in vitro* results concerning the antiproliferative action of 18 recently synthetized solanidine analogs. Some of them proved able to increase the hypodiploid population after a 24-h incubation, which is generally regarded as a marker of programmed cell death, corresponding to the apoptosis-inducing capacity of these steroids. The mechanism of the antiproliferative action was approached through a set of additional *in vitro* experiments. 

During recent years, a number of naturally occurring and synthetic compounds with cytotoxic properties have been reported to interfere with normal DNA topoisomerase functions [25,26]. These enzymes regulate conformational changes in DNA topology by making transient breakages at nucleic acid backbone and rejoining of DNA strands. The identification of new compounds with the ability to interfere with the catalytic cycle of topoisomerases and to stabilize the covalent complex has therefore been recognized as an effective approach for the development of chemotherapeutic agents. We biochemically addressed the potential interference of the compounds on DNA topoisomerase reactions via plasmid DNA relaxation assay. Since the plasmid supercoil relaxation assay results agreed well with those of cell cycle analyses, topoisomerase I can be regarded as potential target of the tested compounds. Indeed, given the crucial roles of DNA topoisomerases in replication, transcription and recombination a considerable number of pharmaceutically important compounds were reported to target these enzymes [25,26]. Further experiments were performed by means of high-throughput, nanocapillary QRT-PCR. Low sample volumes (33 nL in each well), large numbers of reaction chambers, 48 individually addressable submatrices and software-controlled data processing and analysis make this system ideal for our screening purposes. This QRT-PCR has a capacity of around 18000 reactions per day and is optimal for analyzing numerous samples over 56-112 gene markers. We earlier demonstrated the utility of a high-throughput, nanocapillary QRT-PCR system (OpenArray™ Cycler from Applied Biosystems, previously BioTrove) for drug-induced gene expression fingerprinting and *in vivo* toxicogenomics purposes [27,28]. The results of the present assays indicated that the steroids analyzed in the present study exhibited their effects on cell cycle-related genes only 24 h after treatment, an observation in good concordance with our observations on their cytotoxic effects. Two analogs (**10** and **16**) behaved differently, *i.e.*, showed changed gene expression profiles but both compounds repressed the expression of CDKN2B dramatically after one day of incubation, which could at least partially explain their roles in inhibiting cell cycle progression in cancer cells [29]. The differential repression of two cyclins (cyclin E2 and cyclin D1, respectively) warrants further study. Both genes coding for cyclins possess cyclin-dependent protein kinase regulator activity. Cell cycle progression is a highly ordered and tightly-regulated process that involves multiple checkpoints for the assessment of extracellular growth signals, cell size and DNA integrity. Both cyclin E2 and cyclin D1 are positive regulators or accelerators that induce cell cycle progression; whereas inhibition of their abnormal expression could result in anticancer effects [30,31]. Only the active compound **10** dramatically decreased the expression of one of the key players regulating cell cycle progression, the F-box protein SKP2 after a 24-h treatment, which could partly explain its cytotoxic effect.

Besides the investigation of the direct antiproliferative action of the current set of solanidine analog, their MDR-reversal activities were additionally tested. Since more than half of the analogs caused an accumulation of rhodamine-123 comparable to or more pronounced than that of the reference agent verapamil, the solanidine-related skeleton can be regarded as a suitable basis for further ABC transporter inhibitors. Three of the analogs (compounds **3**, **4** and **5**) substantially potentiated the cytostatic action of doxorubicin against lymphoma cells expressing the human ABCB1 transporter. Since compounds **3** and **4** exhibited substantial intrinsic activities their action obtained in combination experiments should not be explained by their effects on the ABC transporter. Compound **5** is free of intrinsic antiproliferative activity; the advantageous interaction may therefore be attributed to its MDR-reversal potency. The presented results revealed that substituted androstene-fused arylpyrazolines exhibit an attractive array of actions, including antiproliferative, apoptosis-inducing and ABC transporter inhibiting-capacities, making this skeleton attractive for the design and synthesis of further innovative drug candidates.

## 3. Experimental

### 3.1. Cancer Cell Lines

Human adenocarcinoma cells (HeLa, purchased from the European Collection of Cell Cultures, Salisbury, UK) were maintained in minimal essential medium supplemented with 10% fetal bovine serum, 1% non-essential amino acids and an antibiotic-antimycotic mixture. A mouse lymphoma cell line (L5178) transfected with the human *MDR1* gene was used for the measurement of MDR reversal. The T-cell line lymphoma cells were transfected with the pHa MDR1/A retrovirus as described by Pastan *et al*. [32,33]. The sensitive (denoted PAR for parental) and the resistant (denoted MDR) cells were maintained in McCoy’s 5A medium supplemented with 10% heat-inactivated horse serum, 1% l-glutamine and an antibiotic-antimycotic combination. All media and supplements were obtained from Life Technologies (Paisley, Scotland, UK). The medium of the MDR cells contained colchicine (60 ng/mL) to ensure P-gp activity. All cell types were cultured at 37 °C in a humidified CO_2_ incubator. Chemicals, if otherwise not specified, were purchased from Sigma-Aldrich Ltd. (Budapest, Hungary).

### 3.2. Cell Cycle Analysis

Flow the determination of cellular DNA content, flow cytometric analysis was used [34]. HeLa cells were treated (180,000/condition) and trypsinized after a 24-h exposure, washed with PBS and permeabilized by detergent treatment (0.1% Triton X-100 in PBS) for 30 min. DNA was then stained with propidium iodide (10 µg/mL) in the presence of RNase (50 µg/mL). The samples were analyzed by FACStar (Becton-Dickinson, Mountain View, CA, USA). In each analysis, 20000 events were recorded, and the percentages of the cells in the different cell cycle phases (subG1, G1, S and G2/M) were calculated by means of winMDI 2.9. Three independent experiments were performed and average values were calculated. The subG1 fractions were regarded as the apoptotic cell population [35].

### 3.3. Plasmid Supercoil Relaxation Assays

Plasmid supercoil relaxation assays were carried out as described previously [36]. Briefly, 20 µL of reaction mixture contained one unit of calf thymus topoisomerase I (the activity removing the supercoils from 500 ng of sc plasmid substrate in 30 mins at 37 °C), 500 ng of supercoiled (sc) pBR322 (Takara, Otsu-Shiga, Japan),in the presence or absence of the 0.1 volume of test compounds in reaction buffer (35 mM Tris-HCl (pH 8.0), 72 mM KCl, 5 mM MgCl_2_, 5 mM DTT, 5 mM spermidine, and 0.1% bovine serum albumin). The relaxation products were separated on 1% agarose gels in TBE buffer (45 mM Tris borate and 1 mM EDTA, pH 8.0) in a horizontal electrophoresis apparatus (5 V/cm) (Thermo EC250) and photographed under UV light after staining in ethidium bromide (EtdBr) solution (0.5 μg/mL). The relationship between the binding of EtdBr and the amount of fluorescence given by sc and relaxed DNA (rlx DNA) under UV light was carried out as described earlier [37]. DNA bands were quantified from gel photo images using BioRad Multianalyst (ver. 1.1); average band intensities were calculated from 3 independent reactions.

### 3.4. RNA Isolation, cDNA Conversion

RNA isolation from cells and cDNA synthesis were performed as published previously [27]. Briefly, cells (4 × 10^6^) were washed with PBS, then lyzed in RA1 buffer (Machery-Nagel, Bethlehem, PA, USA). Total RNA was purified from drug-treated and control cells with AccuPrep™ RNA purification kit (Bioneer, Daeleon, Korea) according to the manufacturer’s protocol, except that DNase I treatment was incorporated. RNA was eluted with 50 µL RNase-free water at 55 °C. The quality and quantity were assessed spectrophotometrically (Nanodrop, Wilmington, DE, USA) and considered accepable if the absorption ratio for 260 nm/280 nm was >1.8. After addition of RNase inhibitor, samples were stored at −80 °C. For QRT-PCR, total RNA (750 ng) was converted into cDNA with the High-Capacity cDNA RT Kit (Applied Biosystems, Foster City, CA, USA) and without purification the mixture was diluted with RNase-free water and subjected to nanocapillary QRT-PCR analysis.

### 3.5. Profiling of RNAs with High-Throughput, nanOcapillary QRT-PCR

Amplification of the samples was followed in real time with an OpenArray NT Cycler (BioTrove Inc., Woburn, MA, USA/Applied Biosystems) as previously described [28,38]. For our gene set related to the cell cycle and apoptosis, individual SybrGreen assays were specified (for genes, functional classes and primer sequences, see Supplementary Table 1). Primer probes were synthetized at BioTrove for loading in their OpenArray plates in a customer-specified layout. The reverse transcribed samples (or water for no template controls) were added to a 384-well plate containing GenAmp Fast PCR Master Mix (Applied BioSystems) and OpenArray DLP 5x Remix Solution (BioTrove Inc.) for OpenArray amplification. The OpenArray autoloader transfered the cDNA/master mix from the plate to the array through-holes by capillary action. Each subarray was loaded with 5.0 μL of Master Mix containing 1.2 μL of reverse transcribed cDNA. The array was manually transferred to the OpenArray slide case and sealed. The plates were cycled in the OpenArray NT cycler under the following conditions: 50 °C for 15 s, 91 °C for 10 min, followed by 50 cycles of 54 °C for 170 s and 92 °C for 45 s. The Biotrove OpenArray NT Cycler System software (version 1.0.2) was used with a proprietary calling algorithm that estimates the quality of each individual threshold cycle (CT) value by calculating a CT confidence value for the amplification reaction. In our assay, CT values with CT confidence values < 300 (average CT confidence of the non-target amplification reactions plus 3 standard deviations) were considered background signals. CT confidence levels higher than 300 were considered positive and were analyzed further. Normalization was done by using the average CT values of four house-keeping genes (tubulin beta 2A, beta-actin, HPRT, and serpin peptidase inhibitor clade B) and gene expression changes were calculated from the averages of four replicate experiments. Average values were accepted when the STD was <0.5 times the average.

### 3.6. Rhodamine-123 Exclusion Assay

The MDR-reversal effect was investigated by rhodamine-123 exclusion assay with a flow cytometer on L5178 mouse lymphoma cells transfected with the human MDR1 gene and its parent (PAR), drug sensitive cells [33,39]. A total of 10^6^/mL cells were incubated with the tested compounds (final concentration: 40 or 400 µM) for 10 min and 10 µL of the substrate rhodamine-123 (1 mg/mL) was added to the samples. Cells were incubated for 20 min at 37 °C, washed twice and resuspended in 0.5 mL of PBS for flow cytometric analysis with a FACStar (Becton-Dickinson). Verapamil (final concentration 40.6 µM) was used as a reference compound. The results of 3 independent tests were calculated for the treated MDR and PAR cell lines as compared with the untreated cells. The fluorescence activity ratio was calculated as the ratio of the fluorescence of the treated and untreated cells.

### 3.7. Antiproliferative Assay

The antiproliferative effects of the solanidine analogs were determined in MTT ([3-(4,5-dimethylthiazol-2-yl)-2,5-diphenyltetrazolium bromide]) assays [40]. Briefly, mouse lymphoma cells were seeded into 96-well plates at a density of 10,000/well and exposed to the tested agents for 72 h. After the incubation period, 20 μL of MTT solution was added and the precipitated formazan crystals were solubilized by the addition of 100 µL of 10% sodium dodecylsulfate. Finally, the absorbance was read at 545 nm with an ELISA reader. Two independent experiments were carried out with at least five parallel experiments, and doxorubicin Ebewe Pharma GmbH (Unterach, Austria) was used as a reference drug.

## 4. Conclusions

A set of solanidine analogs **1**–**18** was recently synthetized from pregnadienolone acetate. These compounds proved to increase the hypodiploid population of HeLa cells, indicating their apoptosis-inducing character. One of the most potent analogs (compound **10**) down-regulated the expression of cell cycle-related genes at the mRNA level including TNFα and SKP2 and induced GADD45alpha. The inhibition of topoisomerase I could be a substantial element of the detected antiproliferative action. Moreover, the MDR-reversal capacities of the novel compounds were evidenced by the accumulation of rhodamine-123 in an ABCB1 transporter-expressing lymphoma cell line. 

## Supplementary Materials

The list of the selected genes, together with their functional categories and primer sequences can be accessed at: http://www.mdpi.com/1420-3049/19/2/2061/s1.
