# Supplementary material for: Anticancer and Multidrug Resistance-Reversal Effects of Solanidine Analogs Synthetized from Pregnadienolone Acetate
Source: Molecules. 2014 Feb 17;19(2):2061–76. doi: 10.3390/molecules19022061 (PMC6271930; doi:10.3390/molecules19022061)
Supplement: Supplementary file 1 [file molecules-19-02061-s001.pdf]

Supplementary Materials

Table S1. The list of the selected genes, together with their functional categories and primer sequences.

Gene#	RefSeqID	FxnlCat	GeneName	Forward	Reverse
1	NM_033379	cyclin-dependent protein kinase activity	CDC2; cell division cycle 2_ G1 to S and G2 to M	aatggaaaccaggaagcctagc	agaaattcgtttggctggatcat
2	NM_001798	cyclin-dependent protein kinase activity	CDK2; cyclin-dependent kinase 2	agactttggactagccagagc	gcagcccaggaggatttcag
3	NM_014326	calmodulin regulated protein kinase activity	DAPK2; death-associated protein kinase 2	gctcactttgatctcaagcca	aaattccggcgtcccaaaaat
4	NM_001348	calmodulin regulated protein kinase activity	DAPK3; death-associated protein kinase 3	ggaggacgtggaggaccatta	ttggctgcgtactccttgc
5	NM_003330	glutathione-disulfide reductase activity	TXNRD1; thioredoxin reductase 1	ctaaggaggcagcccaatatg	gtatgcaacccacattcacaca
6	NM_002423	metalloendopeptidase activity	matrix metallopeptidase 7 (matrilysin uterine)	gaaacacgctggctcatgc	aggaatgtcccatacccaaaga
7	NM_057749	cyclin-dependent protein kinase regulator activity	cyclin E2	tgcttacgtcactgatggtgc	ccaggagatgattgttacaggac
8	NM_032989	structural constituent of ribosome	BCL2-antagonist of cell death	atgagtgacgagtttgtggac	gatcccaccaggactggaaga
9	NM_000389	cyclin-dependent protein kinase inhibitor activity	cyclin-dependent kinase inhibitor 1A (p21 Cip1)	gagactctcagggtcgaaaacg	cttcctgtgggcggattagg
10	NM_001924	DNA repair	growth arrest and DNA-damage-inducible 45 alpha	tggtgacgaatccacattcatc	acttcagtgcaatttggttcagt
11	NM_004064	transforming growth factor beta receptor_ cytoplasmic mediator activity	CDKN1B; cyclin-dependent kinase inhibitor 1B (p27_ Kip1)	ACAAAAGAGCCAACAGAACAG	AGGATGTCCATTCCATGAAG
12	NM_000633	humoral immune response	BCL2; B-cell CLL/lymphoma 2	ggacaacatcgccctgtgg	agccaggagaaatcaaacagag
13	NM_007297	DNA binding	BRCA1; breast cancer 1_ early onset	GAAGACTGCTCAGGGCTATC	TTTCCTGCTGGAGCTTTATC
14	NM_005228	protein serine/threonine kinase activity	EGFR; epidermal growth factor receptor	tgtgcaacgtggagagcatc	gcttggatcacacttttggcag
15	NM_001964	transcription factor activity	EGR1; early growth response 1	cctgaccgcagagtcttttcc	cggccagtataggtgatggg
16	NM_002133	heme oxygenase (decyclizing) activity	HMOX1; heme oxygenase (decycling) 1	gagggtgatagaagaggccaa	tgaggggctctggtccttg
17	NM_002592	leading strand elongation	proliferating cell nuclear antigen	ACTAAAATGCGCCGGCAAT	TCTCCTGGTTTGGTGCTTCAA
18	NM_032991	cysteine-type peptidase activity	CASP3_ caspase 3_ apoptosis-related cysteine peptidase	CTGGACTGTGGCATTGAGACA	TCGGCCTCCACTGGTATTTT
19	NM_000963	prostaglandin-endoperoxide synthase activity	PTGS2; prostaglandin-endoperoxide synthase 2	ctagagcccttcctcctgtg	ggggatcagggatgaactttctt
20	NM_000546	nuclease activity	TP53; tumor protein p53 (Li-Fraumeni syndrome)	gtgctttccacgacggtga	ggctcgacgctaggatctg
21	NM_005427	transcription factor activity	TP73; tumor protein p73	tgcctgtttacaagaaagcgg	gctggagcagactgtccttc
22	NM_000660	transforming growth factor beta receptor binding	TGFB1; transforming growth factor_ beta 1	TACTACGCCAAGGAGGTCAC	GGAGCTCTGATGTGTTGAAG
23	NM_000594	tumor necrosis factor receptor binding	TNF; tumor necrosis factor (TNF superfamily_ member 2)	tgtagcaaaccctcaagctga	cctctgatggcaccaccag
24	NM_003844	death receptor activity	TNFRSF10A; tumor necrosis factor receptor superfamily_ member 10a	atgggaacatagccctttggg	caaattgttggaagcattggtgt
25	NM_003842	death receptor activity	TNFRSF10B; tumor necrosis factor receptor superfamily_ member 10b	tgtcccagagggatggtcaag	ggtcaccgtctcctccaca
26	NM_001066	tumor necrosis factor receptor activity	TNFRSF1B; tumor necrosis factor receptor superfamily_ member 1B	cccaggtggcatttacaccc	tggtacagaagacttttgcatgt
27	NM_003327	tumor necrosis factor receptor activity	TNFRSF4; tumor necrosis factor receptor superfamily_ member 4	ccgggcttctacaacgacg	cagactgtgtcctgtgtggc
28	NM_001455	transcription factor activity	FOXO3A; forkhead box O3A	ggctggaagaactccatccg	cagggttgatgatccaccaag
29	NM_004104	fatty acid metabolism	FASN; fatty acid synthase	cagattcactccgaggaacac	gccctctgaagtcgaagaagaa
30	NM_001749	calpain activity	CAPNS1; calpain_ small subunit 1	cgacgctactcagatgaaagtg	gggctccagttcaggaatacat
31	NM_001239	cyclin-dependent protein kinase activity	CCNH; cyclin H	tcacttgtgcatttttggcct	gtgccttctcctgtccaagag
32	NM_001240	transcription from Pol II promoter	CCNT1; cyclin T1	tcacacagttccctggaaattc	gatggagacaagtatgtgctacc
33	NM_001241	transcription from Pol II promoter	CCNT2; cyclin T2	gatcaccattgaacacccaca	ggttgtaagatgcagactgttgg
34	NM_003592	protein binding	CUL1; cullin 1	accacagagatgcgggtttg	ccagtgcagcaatccttattca
35	NM_001168	receptor activity	BIRC5; baculoviral IAP repeat-containing 5 (survivin)	cttggcccagtgtttcttctg	ggctctttctctgtccagtttc
36	NM_013366	ubiquitin-dependent protein catabolism	APC2; anaphase-promoting complex subunit 2	CTGTCATCCTGTCCAGTGAG	TGGCTTGGTCCTGAAAATAC
37	NM_013367	ubiquitin-dependent protein catabolism	APC4; anaphase-promoting complex subunit 4	cagtagaaagcagtgttctcaca	agagcttcctccaaggacgag
38	NM_053056	cyclin-dependent protein kinase regulator activity	CCND1; cyclin D1 (PRAD1: parathyroid adenomatosis 1)	aggaacagaagtgcgaggag	ccacgaacatgcaagtggc
39	NM_002388	chromatin binding	MCM3; MCM3 minichromosome maintenance deficient 3	GCCAGGACATCTCCAGTTAC	TGGCTTTTCTCCTCTTCATC

Table S1. *Cont.*

**Gene#**	**RefSeqID**	**FxnlCat**	**GeneName**	**Forward**	**Reverse**
40	NM_006739	chromatin binding	MCM5; minichromosome maintenance deficient 5_ cell division cycle 46	gtgctccaggacatccagg	gatgatgccagggatcttcac
41	NM_004507	DNA repair	HUS1; HUS1 checkpoint homolog (S. pombe)	ggtcccagatcctgatgttagt	gggtgctttcagaggctaatg
42	NM_133341	DNA repair	RAD17; RAD17 homolog (S. pombe)	gaagtgcaataaacagcctcca	tgacagcacagcatctgatttt
43	NM_000077	transcription factor activity	CDKN2A; cyclin-dependent kinase inhibitor 2A	cgacttcaggtgtgccacatt	ctcaaatcctctggagggacc
44	NM_078487	transcription factor activity	CDKN2B; cyclin-dependent kinase inhibitor 2B	acggagtcaaccgtttcgg	gcgatctaggttccagccc
45	NM_003334	ubiquitin activating enzyme activity	UBE1; ubiquitin-activating enzyme E1	tgtcactgcctacactggac	gacagaactcacccactcgc
46	NM_007313	protein-tyrosine kinase activity	ABL1; v-abl Abelson murine leukemia viral oncogene homolog 1	gcttcttggtgcgtgagagt	aggagacgtagagcttgcca
47	NM_001826	cyclin-dependent protein kinase activity	CKS1B; CDC28 protein kinase regulatory subunit 1B	agagcgatcatgtcgcacaaa	accagcttggctatgtccttg
48	NM_001827	cyclin-dependent protein kinase activity	CKS2; CDC28 protein kinase regulatory subunit 2	aggagtggaggagacttggtg	ttggaagaggtcgtctaaagaga
49	NM_000059	single-stranded DNA binding	BRCA2; breast cancer 2_ early onset	caccacccacccttagttcta	tgacttgcagcttctctttgatt
50	NM_005983	ubiquitin-dependent protein catabolism	SKP2; S-phase kinase-associated protein 2 (p45)	gtttgtaagaggtggtatcgcc	agcaaccgaccagtcacatc
51	NM_030655	DNA helicase activity	DDX11; DEAD/H (Asp-Glu-Ala-Asp/His) box polypeptide 11	cttcacaccctattccatccagg	caaagtcacggagccaagaga
52	NM_005192	Protein tyrosine/serine/threonine phosphatase activity	CDKN3; cyclin-dependent kinase inhibitor 3	actgctatggaggacttggga	ctcgcaggctgtctatggc
53	NM_001069	control	tubulin beta 2A	AGACCGCATCATGAACACCTT	GGAGTAGGTTTCATCTGTGTTTTCC
54	NM_001101	control	Beta-Actin	aaccgcgagaagatgaccc	atcacgatgccagtggtacg
55	NM_000194	control	HPRT	actggcaaaacaatgcagact	caacacttcgtggggtcct
56	NM_002639	control	serpin peptidase inhibitor clade B (ovalbumin) member 5	CCAAACCAGTGCAGATGATGAA	GGATGAACATGCTGAGATGCTTATT
57		negative control	empty		
58		negative control	empty		
59		negative control	empty		
60		negative control	empty		
61		negative control	empty		
62		negative control	empty		
63		negative control	empty		
64		negative control	empty		
